# Gene-Centric Meta-Analysis of Lipid Traits in African, East Asian and Hispanic Populations

**DOI:** 10.1371/journal.pone.0050198

**Published:** 2012-12-07

**Authors:** Clara C. Elbers, Yiran Guo, Vinicius Tragante, Erik P. A. van Iperen, Matthew B. Lanktree, Berta Almoguera Castillo, Fang Chen, Lisa R. Yanek, Mary K. Wojczynski, Yun R. Li, Bart Ferwerda, Christie M. Ballantyne, Sarah G. Buxbaum, Yii-Der Ida Chen, Wei-Min Chen, L. Adrienne Cupples, Mary Cushman, Yanan Duan, David Duggan, Michele K. Evans, Jyotika K. Fernandes, Myriam Fornage, Melissa Garcia, W. Timothy Garvey, Nicole Glazer, Felicia Gomez, Tamara B. Harris, Indrani Halder, Virginia J. Howard, Margaux F. Keller, M. Ilyas Kamboh, Charles Kooperberg, Stephen B. Kritchevsky, Andrea LaCroix, Kiang Liu, Yongmei Liu, Kiran Musunuru, Anne B. Newman, N. Charlotte Onland-Moret, Jose Ordovas, Inga Peter, Wendy Post, Susan Redline, Steven E. Reis, Richa Saxena, Pamela J. Schreiner, Kelly A. Volcik, Xingbin Wang, Salim Yusuf, Alan B. Zonderland, Sonia S. Anand, Diane M. Becker, Bruce Psaty, Daniel J. Rader, Alex P. Reiner, Stephen S. Rich, Jerome I. Rotter, Michèle M. Sale, Michael Y. Tsai, Ingrid B. Borecki, Robert A. Hegele, Sekar Kathiresan, Michael A. Nalls, Herman A. Taylor, Hakon Hakonarson, Suthesh Sivapalaratnam, Folkert W. Asselbergs, Fotios Drenos, James G. Wilson, Brendan J. Keating

**Affiliations:** 1 Department of Genetics, University of Pennsylvania, School of Medicine, Philadelphia, Pennsylvania, United States of America; 2 Department of Medical Genetics, Biomedical Genetics, University Medical Center, Utrecht, The Netherlands; 3 Julius Center for Health Sciences and Primary Care, University Medical Center Utrecht, Utrecht, The Netherlands; 4 Center for Applied Genomics, Abramson Research Center, The Children's Hospital of Philadelphia, Philadelphia, Pennsylvania, United States of America; 5 BGI-Shenzhen, Shenzhen, People's Republic of China; 6 Department of Cardiology, Division Heart and Lungs, University Medical Center Utrecht, Utrecht, The Netherlands; 7 Department of Clinical Epidemiology, Biostatistics and Bioinformatics, Academic Medical Center, University of Amsterdam, Amsterdam, The Netherlands; 8 Departments of Medicine and Biochemistry, Schulich School of Medicine and Dentistry, University of Western Ontario, London, Ontario, Canada; 9 Center for Public Health Genomics, University of Virginia, Charlottesville, Virginia, United States of America; 10 GeneSTAR Research Program, Division of General Internal Medicine, Johns Hopkins School of Medicine, Baltimore, Maryland, United States of America; 11 Department of Biostatistics, University of Alabama at Birmingham, Birmingham, Alabama, United States of America; 12 Baylor College of Medicine, Houston, Texas, United States of America; 13 Jackson Heart Study, Jackson State University, Jackson, Mississippi, United States of America; 14 Medical Genetics Institute, Cedars-Sinai Medical Center, Los Angeles, California, United States of America; 15 Department of Public Health Sciences, University of Virginia, Charlottesville, Virginia, United States of America; 16 Boston University, Boston, Massachusetts, United States of America; 17 The National Heart, Lung, Blood Institute's Framingham Heart Study, Framingham, Massachusetts, United States of America; 18 Division of Statistical Genomics and Department of Genetics Washington University School of Medicine, St. Louis, Missouri, United States of America; 19 Translational Genomics Research Institute, Phoenix, Arizona, United States of America; 20 The University of Texas Health Science Center at Houston, Houston, Texas, United States of America; 21 Laboratory for Epidemiology, Demography, and Biometry, National Institute on Aging, National Institutes of Health, Bethesda, Maryland, United States of America; 22 School of Medicine, University of Pittsburgh, Pittsburgh, Pennsylvania, United States of America; 23 Division of Public Health Sciences, Fred Hutchinson Cancer Research Center, Seattle, Washington, United States of America; 24 Department of Preventive Medicine, University of Tennessee Health Science Center, Memphis, Tennessee, United States of America; 25 Sticht Center on Aging, Wake Forest University, Winston-Salem, North Carolina, United States of America; 26 Department of Preventive Medicine, Northwestern University Feinberg School of Medicine, Chicago, Illinois, United States of America; 27 Department of Epidemiology and Prevention, Division of Public Health Sciences, Wake Forest University, Winston-Salem, North Carolina, United States of America; 28 Department of Human Genetics, University of Pittsburgh, Pittsburgh, Pennsylvania, United States of America; 29 Broad Institute, Cambridge, Massachusetts, United States of America; 30 Harvard Medical School, Boston, Massachusetts, United States of America; 31 Massachusetts General Hospital, Boston, Massachusetts, United States of America; 32 Department of Epidemiology, University of Pittsburgh, Pittsburgh, Pennsylvania, United States of America; 33 JM-USDA Human Nutrition Research Center on Aging at Tufts University, Boston, Massachusetts, United States of America; 34 Department of Genetics and Genomic Sciences, Mount Sinai School of Medicine, New York, New York, United States of America; 35 Division of Cardiology, Department of Medicine, Johns Hopkins University, Baltimore, Maryland, United States of America; 36 Brigham and Women's Hospital and Beth Israel Deaconess Medical Center, Harvard Medical School, Boston, Massachusetts, United States of America; 37 Center for Human Genetic Research, Massachusetts General Hospital, Boston, Massachusetts, United States of America; 38 Program in Medical and Population Genetics, Broad Institute, Cambridge, Massachusetts, United States of America; 39 School of Public Health, University of Minnesota, Minneapolis, Minnesota, United States of America; 40 Division of Epidemiology, Human Genetics and Environmental Sciences, Human Genetics Center, School of Public Health, University of Texas Health Science Center, Houston, Texas, United States of America; 41 Population Health Research Institute, Hamilton Health Sciences, McMaster University, Hamilton, Ontario, Canada; 42 Cardiovascular Health Research Unit, Departments of Medicine, Epidemiology, and Health Services, University of Washington, Seattle, Washington, United States of America; 43 Group Health Research Institute, Group Health Cooperative, Seattle, Washington, United States of America; 44 Cardiovascular Institute, the Perelman School of Medicine at the University of Pennsylvania, Philadelphia, Pennsylvania, United States of America; 45 Department of Biochemistry and Molecular Genetics, University of Virginia, Charlottesville, Virginia, United States of America; 46 Department of Medicine, University of Virginia, Charlottesville, Virginia, United States of America; 47 Department of Laboratory Medicine and Pathology, University of Minnesota, Minneapolis, Minnesota, United States of America; 48 Robarts Research Institute, University of Western Ontario, London, Ontario, Canada; 49 Laboratory of Neurogenetics, Intramural Research Program, National Institute on Aging, National Institutes of Health, Bethesda, Maryland, United States of America; 50 Jackson State University, Tougaloo College, and the University of Mississippi Medical Center, Jackson, Mississippi, United States of America; 51 Department of Vascular Medicine, Academic Medical Center, Amsterdam, The Netherlands; 52 Centre for Cardiovascular Genetics, Institute of Cardiovascular Science, Faculty of Population Health Sciences, University College London, London, United Kingdom; 53 Department of Physiology and Biophysics, University of Mississippi Medical Center, Jackson, Mississippi, United States of America; 54 Department of Medicine, Thrombosis and Hemostasis Program, University of Vermont, Burlington, Vermont, United States of America; 55 Division of Endocrinology, Diabetes and Medical Genetics, College of Medicine, Medical University of South Carolina, Charleston, SC United States of America; 56 Department of Nutrition Sciences, University of Alabama at Birmingham, Birmingham, Alabama, United States of America; 57 Department of Epidemiology, University of Alabama at Birmingham, Birmingham, Alabama, United States of America; 58 Laboratory of Personality and Cognition, National Institute on Aging, National Institutes of Health, Bethesda, Maryland, United States of America; 59 Health Disparities Unit, National Institute on Aging, National Institutes of Health, Baltimore, Maryland, United States of America; 60 Heart and Vascular Institute, School of Medicine, University of Pittsburgh, Pittsburgh, Pennsylvania, United States of America; 61 School of Health Sciences, Department of Epidemiology and Biostatistics, Jackson State University, Jackson, Mississippi, United States of America; College of Pharmacy, University of Florida, United States of America

## Abstract

Meta-analyses of European populations has successfully identified genetic variants in over 100 loci associated with lipid levels, but our knowledge in other ethnicities remains limited. To address this, we performed dense genotyping of ∼2,000 candidate genes in 7,657 African Americans, 1,315 Hispanics and 841 East Asians, using the IBC array, a custom ∼50,000 SNP genotyping array. Meta-analyses confirmed 16 lipid loci previously established in European populations at genome-wide significance level, and found multiple independent association signals within these lipid loci. Initial discovery and *in silico* follow-up in 7,000 additional African American samples, confirmed two novel loci: rs5030359 within *ICAM1* is associated with total cholesterol (TC) and low-density lipoprotein cholesterol (LDL-C) (*p* = 8.8×10^−7^ and *p* = 1.5×10^−6^ respectively) and a nonsense mutation rs3211938 within *CD36* is associated with high-density lipoprotein cholesterol (HDL-C) levels (*p* = 13.5×10^−12^). The rs3211938-G allele, which is nearly absent in European and Asian populations, has been previously found to be associated with CD36 deficiency and shows a signature of selection in Africans and African Americans. Finally, we have evaluated the effect of SNPs established in European populations on lipid levels in multi-ethnic populations and show that most known lipid association signals span across ethnicities. However, differences between populations, especially differences in allele frequency, can be leveraged to identify novel signals, as shown by the discovery of *ICAM1* and *CD36* in the current report.

## Introduction

Plasma levels of circulating total cholesterol (TC), low-density lipoprotein (LDL-C), high-density lipoprotein (HDL-C) and triglycerides (TG) are associated with coronary artery disease (CAD) and are targets for therapeutic intervention [1]. Multiple environmental and genetic factors influence these plasma lipid levels, with heritability estimated to range from 0.28 to 0.78 in twin and family studies [2]. To date, >100 lipid-associated loci have been described, using studies mainly based on individuals of European ancestry [3]. Together, known variants affecting plasma lipid levels explain 10–12% of the total variance and 25–30% of the genetic variance [3] indicating that other loci and independent signals in established loci are likely to additionally contribute to the trait.

Lipid levels have been demonstrated to vary between ethnic groups [4]. Africans and East Asians have higher levels of HDL-C and lower levels of TG compared to Europeans [5] though the underlying mechanisms of these ethnic differences remain unknown. Genetic contributors to lipid concentrations are less well understood in non-European populations partly due to less well-powered genetic studies being attempted to date and most genotyping platforms are designed to have optimal coverage in European studies. An important first step towards understanding genetic risk across populations is to establish whether plasma lipid associated loci, identified in Europeans, span across multiple ethnicities or are population-specific. In a recent analysis, most of these known lipid loci had the same direction of association in different ethnic groups as in Europeans, despite presumed differences in linkage disequilibrium (LD) between marker and causal variants in each population [6]. Using regional LD in different ethnicities can help to refine association signals and to distinguish causal variants from correlated markers [7]. Furthermore, independent association signals in established lipid loci in one ethnicity may be useful to highlight causal signal(s) in other ethnicities.

The ITMAT-Broad-CARe (IBC) array (also referred to as the CardioChip or HumanCVD Beadchip [Illumina]) was specifically designed to densely tag ∼2000 genes with known or potential roles in lipid and cardiovascular traits using ∼50,000 single nucleotide polymorphisms (SNPs) [8]. Sequencing data from European, African American and Yoruba individuals was included for SNP selection in IBC array development. The IBC array drew upon knowledge of lipid metabolism and cardiovascular physiology, as well as early GWAS and sequencing studies to target efforts towards regions with higher *a priori* evidence of association, reducing cost per sample, and improving efficiency of replication studies. The IBC array has been successfully used for multiple cardiovascular-related phenotypes [9], [10], [11], [12]. Results are reported elsewhere for the association of lipid phenotypes in European-derived cohorts with variants on the IBC array [13].

In this study we set out to discover novel lipid loci, fine map signals to identify causal genes at implicated loci, and gain a greater understanding of the genetic architecture of lipid traits across ethnicities. Here, we have used the IBC array to examine association results for TC, LDL-C, HDL-C and TG across seven non-European study populations, including African Americans (n = 7,657), Hispanics (n = 1,315) and East Asians (n = 841). Using conditional analyses, we sought to identify independent signals from within associated loci. Finally, we assessed the direction of effect in non-Europeans of new and established loci found in European-derived populations, and tested a composite risk score of known loci across ethnicities.

## Materials and Methods

### Ethics statement

All participants in each of the cohorts gave informed written consent. The Institutional Review Boards (IRBs) of each CARe cohort (i.e., the IRBs for each cohort's field centers, coordinating center, and laboratory center) have reviewed and approved the cohort's interaction with CARe. The study described in this manuscript was approved by the Committee on the Use of Humans as Experimental Subjects (COUHES) of the Massachusetts Institute of Technology.

### Participating studies

**Figure 1:**
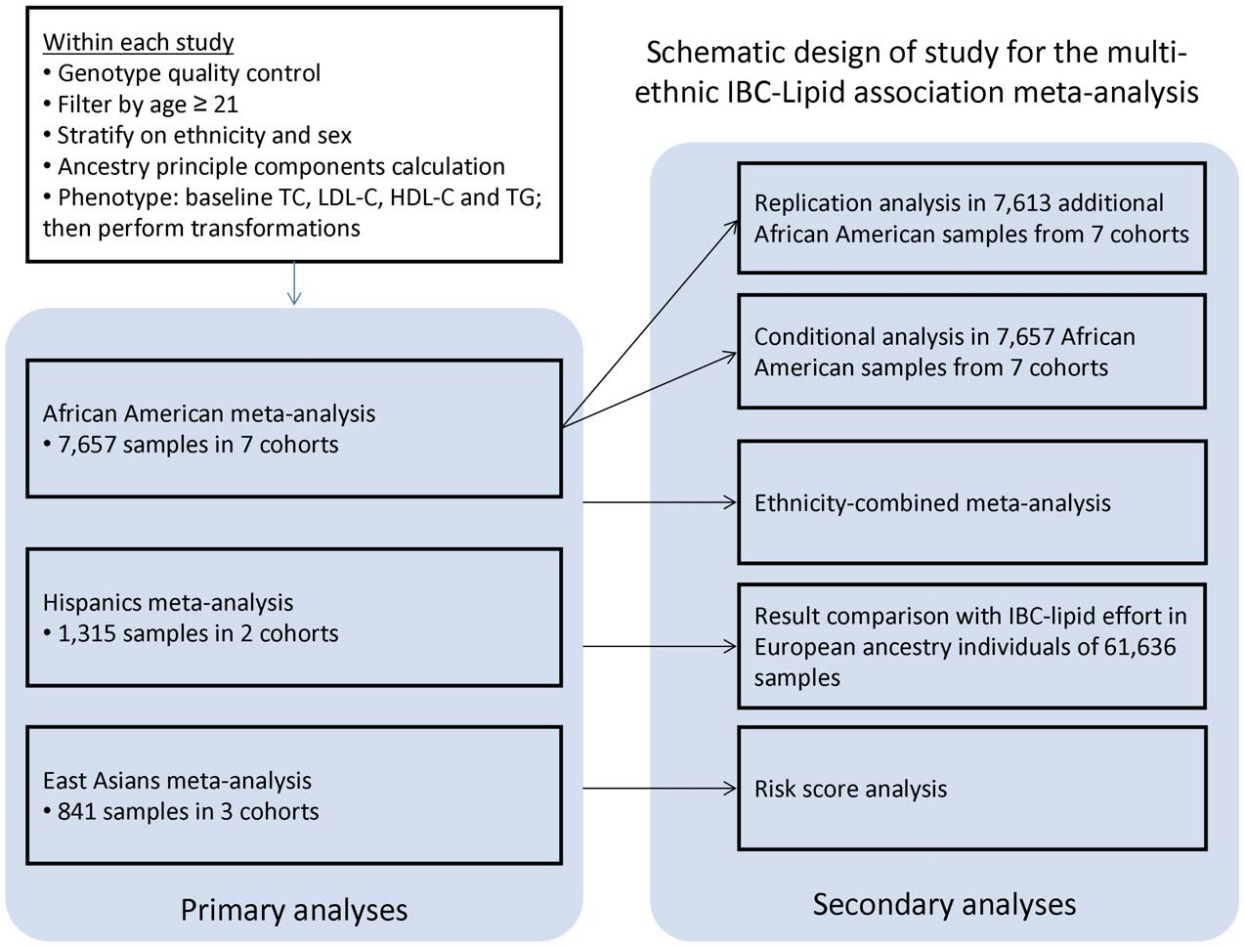
Schematic design of study for the multi-ethnic IBC-Lipid association meta-analysis. The workflow includes primary analyses and secondary analyses. Details can be found in the text.

Data from African-American, Hispanic and East Asian participants from seven cohorts were included for this study (Figure 1). Participants were ≥21 years of age. All seven studies contributed individual-level genotypes and phenotypes. Features of the included cohorts are presented in Table S1 and summary statistics are listed in Table S2. Six replication studies were used comprising African American individuals.

### Phenotype definitions

Lipid phenotypes were taken from baseline or first measurements for all fasting individuals. All measurements were converted to mmol/L, with TC and HDL-C measurements converted from mg/dL by dividing by 38.67, and TG measurements converted from mg/dL by dividing by 88.57. TG values were log(10)-transformed as TG values were not normally distributed. LDL-C was calculated according to Friedewald's formula L∼C – H - kT where C is total cholesterol, H is HDL-C, L is LDL-C, T is TG and k is 0.45 for mmol/L (or 0.20 if measured in mg/dl) [14]. If TG values were >4.51 mmol/L (>400 mg/dL), then LDL-C was treated as a missing value.

### Genotyping and quality control

Genotyping in each participating cohort was performed using the IBC array [8]. SNPs were clustered into genotypes using the Illumina Genomestudio software and were subjected to quality control filters at the sample and SNP level, separately within each cohort. Samples were excluded for individual call rates <90%, gender mismatch, and duplicate discordance. SNPs were removed for call rates <95% or Hardy-Weinberg equilibrium (HWE) p<10^−7^. Due to low frequency SNPs included in the design, and the aim to capture low frequency variants of large effect across the combined dataset, we filtered only on minor allele frequency (MAF)<0.005.

### Statistical analyses

#### Evaluation of population stratification

Self-reported ethnicity was verified by multidimensional scaling analysis of identity-by-state distances as implemented in PLINK [15], including HapMap panels as reference standards. After pruning of SNPs in linkage disequilibrium (r^2^>0.3), Eigenstrat was used to compute principal components within each ethnic group separately for use as covariates in the regression analyses [16].

#### Association testing

Association analysis was performed in each study using an additive genetic model with one degree of freedom. Gender stratified analyses were performed using three multivariate models: Model 1, including 10 principal components (PCs); Model 2, including 10 PCs, age, and lipid medication; and Model 3, including 10 PCs, age, lipid medication, type 2 diabetes (T2D), smoking and BMI. The genomic control inflation factor, lambda, was calculated for each cohort and used for within-study correction before meta-analysis. Genomic control inflation factors (λ) ranged from 1.00 to 1.054.

Meta-analyses within each ethnic group were performed by two independent analysts using a fixed-effect inverse-variance approach in two different software packages: MANTEL (www.broadinstitute.org/~debakker/mantel.html) and METAL [17]. Results were highly concordant, reflecting a robust data analyses pipeline. Additionally, the directions of effect of lead SNPs from previously identified loci from the European IBC array meta-analysis [13] were evaluated for consistency in African Americans, Hispanics and Asians. To gauge an appropriate significance threshold, data from the Candidate gene Association Resource (CARe) IBC array studies [18] which is available on dbGAP (www.ncbi.nlm.nih.gov/gap) were employed and it was determined that after accounting for LD, the effective number of independent tests was ∼26,500 for African Americans, ∼23,500 for Hispanics, and ∼15,500 for East Asians. This produces experimental or ‘array-wide’ statistical thresholds of *p* = 1.9×10^−6^, *p* = 2.1×10^−6^ and *p* = 3.2×10^−6^, respectively, to maintain a false positive rate of 5% for each of the three ethnic groups. While we have adopted these ‘array-wide’ statistical thresholds for this study, we also highlight loci associated at a more conventional genome-wide significance threshold of *p*<5.0×10^−8^.

Additionally, the *I^2^* statistic was calculated to quantify the proportion of total variation due to heterogeneity, as described previously [19].

#### Conditional Analyses

Loci harboring evidence for association of *P*<1×10^−5^ in African Americans were examined for the presence of multiple, independent signals via conditional analyses in PLINK [15]. A term was added to the regression model including the lead SNP as a covariate, and SNPs within a +/−500 kb region were evaluated for significance. A locus-specific Bonferroni correction, as employed in previous IBC studies [20], was applied to determine significance of independent signals within candidate genes genotyped at each locus. On average, the windows contained 195.2 (±107.0) variants with a range between 12 for *ACADL* and 359 for *PCSK9*. Because of limited power due to low sample size, we did not perform conditional analyses in Hispanics and East Asians.

#### Genetic Risk Score Analyses and direction of effect

Within each ethnic group, we generated a genetic risk score using 28 SNPs for TC, 20 SNPs for LDL-C, 24 SNPs for HDL-C, and 21 SNPs for TG that had been found to be array-wide significant (*p* = 2.6×10^−6^) in the European-ancestry IBC meta-analysis [21] (Table S3), weighted by the beta as described previously [22], [23]. To account for missing data we adjusted the values for the number of genotyped risk alleles per individual. We evaluated for each ethnic group the contribution of the weighted genetic risk score to TC, HDL-C, LDL-C and TG in linear regression models adjusting for 10 PCs. Additionally, we compared the relative betas across quartiles of risk by linear regression. These loci were additionally investigated to study direction of effect across ethnicities.

### Replication

In order to confirm putative novel loci, we replicated previously undetected lipid signals (p<1.0×10^−5^) in 7,000 African American individuals from six replication cohorts and in 61,636 samples from the European-ancestry IBC meta-analysis [21]. Recent power analyses suggest that large-scale multi-ethnic association studies may have greater statistical power to detect causal alleles because of random genetic drift elevating global risk variants to higher allele frequency in some populations [24]. All but one replication studie provided summary results of SNPs that were genotyped on platforms other than the IBC array, or imputed using 1000 Genomes data. Features of the replication datasets included in this meta-analysis are described in Table S1.

## Results

### Meta-analyses of African, Hispanic and East Asian populations

**Figure 2:**
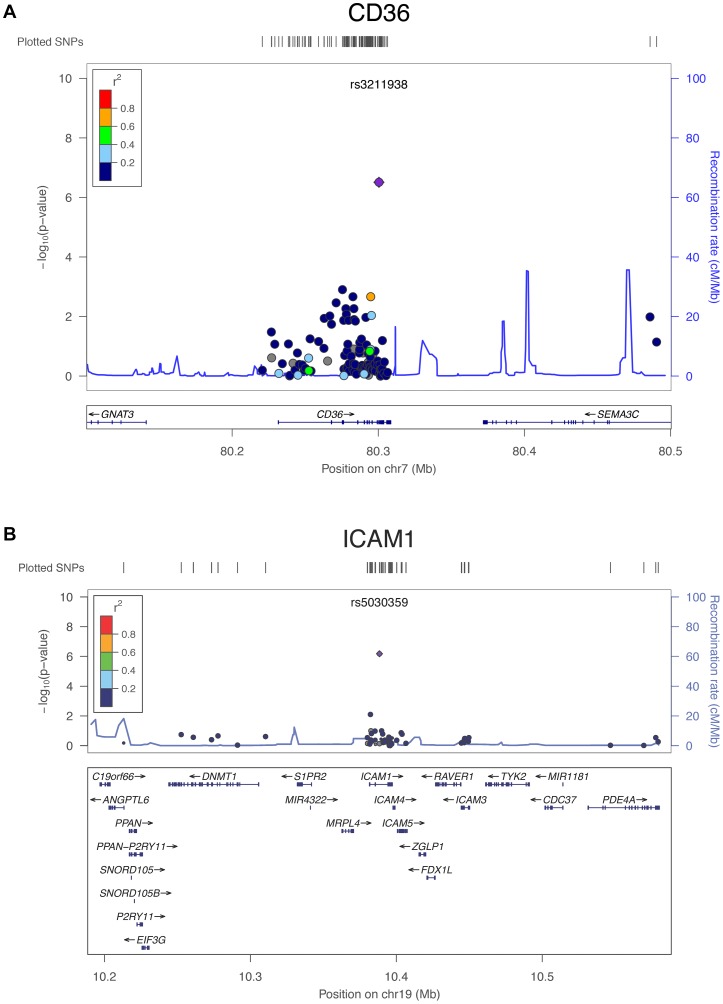
Regional plots for novel lipid loci with array-wide significant regions in IBC meta-analysis of African ancestry. **A.** *CD36* region, **B.** *ICAM1* region. Loci are shown as the lead SNP with a flanking region depicting the candidate gene and nearby genes included on the array. The purple diamond represents the lead SNP in the IBC meta-analysis and the dots represent the surrounding SNPs, with the different colors showing the LD relationship with the lead SNP based on YRI HapMap II information. −log10 p-values for association with HDL-C (for *CD36*) and TC (for *ICAM1*) are shown for each SNP (left-hand axis). Recombination rates in YRI HapMap II is shown in blue traces (right-hand axis).

Meta-analyses of IBC array association results for plasma TC, LDL-C, HDL-C and TG levels in five African American studies (n = 7,657), two Hispanic studies (n = 1,315) and three East Asian studies (n = 841) were performed independently. Results of different association models did not differ substantially. Therefore, results of model 1, an additive model with 10 PCs as covariates, are presented in the main text (Table 1) and results of other models are presented in the supplements (Table S4). After fixed-effect inverse-variance meta-analysis, we found that 23, five and two loci in African Americans, Hispanics and East Asian samples respectively, were significantly associated with a lipid trait at their respective array-wide significance thresholds, with twelve, three and one loci respectively surpassing the traditional genome-wide significance threshold (see Table 1; Figure 1). Two of these loci, intercellular adhesion molecule 1 (*ICAM1*) and CD36 molecule thrombospondin receptor (*CD36*), have not previously been reported to be associated with a lipid trait in a large-scale genomic study (Figure 2).

**Table 1:** Associated loci with lipid traits in individuals of African, East Asian and Hispanic ancestry.

African-Americans										Results IBC Europeans			
Trait	Chr	BP	Candidate Gene	SNP	Risk allele	RAF	Beta (SE)	P	% I2	RAF	Beta (SE)	P	% I2
TC	1	55290748	*PCSK9*	rs11806638	C	0.68	0.11 (0.018)	1.99×10−9	8	0.94	0.01 (0.013)	0.28	0
TC	1	109619113	*CELSR2*	rs12740374	G	0.75	0.14 (0.019)	4.43×10−13	0	0.76	0.13 (0.008)	1.79×10−63	34.5
TC	2	21119200	*APOB*	rs12720826	T	0.87	0.13 (0.025)	7.84×10−8	0	0.999	NA	NA	NA
TC	2	210769915	*ACADL*	rs6739874	T	0.05	0.17 (0.038)	4.65×10−6	0	0.18	NA	NA	NA
TC	19	10249462	*ICAM1*	rs5030359	G	0.99	0.57 (0.098)	5.22×10−9	0	0.998	NA	NA	NA
TC	19	11063306	*LDLR*	rs6511720	G	0.86	0.18 (0.024)	1.39×10−13	26.2	0.88	0.17 (0.009)	1.81×10−73	24.3
TC	19	50112183	*APOE*	rs389261	A	0.25	0.13 (0.020)	2.07×10−11	0	0.001	NA	NA	NA
LDL-C	1	55290528	*PCSK9*	rs11800231	G	0.83	0.14 (0.022)	1.02×10−10	37.3	0.96	0.009 (0.015)	0.60	0
LDL-C	1	109619113	*CELSR2*	rs12740374	G	0.75	0.16 (0.019)	1.92×10−17	0.8	0.75	0.12 (0.007)	2.62×10−66	41.6
LDL-C	2	21141826	*APOB*	rs562338	G	0.4	0.09 (0.017)	6.54×10−8	0	0.82	0.12 (0.008)	7.38×10−51	0
LDL-C	17	61641042	*APOH*	rs1801689	C	0.007	0.49 (0.098)	4.70×10−7	25.5	0.03	0.11 (0.016)	8.57×10−12	0
LDL-C	19	10249462	*ICAM1*	rs5030359	G	0.99	0.51 (0.096)	1.09×10−7	0	0.998	NA	NA	NA
LDL-C	19	11059187	*LDLR*	rs17248720	C	0.73	0.11 (0.019)	7.13×10−10	0	0.88	0.16 (0.009)	8.81×10−72	37.5
LDL-C	19	50112183	*APOE*	rs389261	A	0.25	0.14 (0.020)	1.03×10−12	0	0.001	NA	NA	NA
HDL-C	7	80138385	*CD36*	rs3211938	G	0.09	0.06 (0.012)	3.09×10−7	28.3	0.0005	NA	NA	NA
HDL-C	8	19868772	*LPL*	rs13702	C	0.51	0.04 (0.007)	1.34×10−9	46.8	0.3	0.04 (0.002)	3.57×10−74	24
HDL-C	15	56511231	*LIPC*	rs2070895	A	0.51	0.04 (0.007)	4.16×10−8	0	0.21	0.04 (0.002)	9.76×10−58	41.2
HDL-C	16	55553328	*CETP*	rs17231520	A	0.07	0.19 (0.013)	2.03×10−46	14.5	0.002	0.16 (0.044)	3.28×10−04	0
HDL-C	16	66534352	*LCAT*	rs35673026	T	0.004	0.28 (0.059)	2.40×10−6	0	0.001	NA	NA	NA
TG	2	27584444	*GCKR*	rs1260326	T	0.15	0.06 (0.012)	5.54×10−7	13.7	0.41	0.06 (0.003)	1.56×10−83	32.6
TG	8	19864004	*LPL*	rs328	C	0.93	0.08 (0.016)	1.74×10−7	19.9	0.9	0.08 (0.005)	5.73×10−16	38.1
TG	11	116170289	*APOA5*	rs9804646	C	0.65	0.04 (0.009)	2.55×10−6	2.3	0.92	0.02 (0.005)	1.57×10−7	11.4
TG	19	50114427	*APOE*	rs12721054	A	0.89	0.12 (0.013)	1.01×10−21	2.7	0.998	NA	NA	NA

East Asians										Results IBC Europeans			
Trait	Chr	BP	Candidate Gene	SNP	Risk allele	RAF	Beta (SE)	P	% I2	RAF	Beta (SE)	P	% I2
HDL-C	11	116168917	*ZNF259/APOA5*	rs662799	A	0.71	0.09 (0.018)	8.91×10−7	0	0.93	0.03 (0.004)	1.42×10−18	28.3
TG	11	116168917	*ZNF259/APOA5*	rs662799	G	0.29	0.21 (0.029)	1.57×10−13	28.4	0.07	0.1102 (0.0058)	5.93×10−89	58

Hispanics										Results IBC Europeans			
Trait	Chr	BP	Candidate Gene	SNP	Risk allele	RAF	Beta (SE)	P	% I2	RAF	Beta (SE)	P	% I2
TC	1	109619113	*CELSR2*	rs12740374	G	0.78	0.22 (0.045)	7.88×10−7	41.5	0.76	0.13 (0.008)	1.79×10−63	34.5
TC	12	130896484	*MMP17*	rs10902456	G	0.73	0.19 (0.043)	6.96×10−6	0	0.69	−0.005 (0.006)	0.40	0
LDL-C	1	109619113	*CELSR2*	rs12740374	G	0.78	0.21 (0.040)	1.10×10−7	25.8	0.76	0.13 (0.008)	2.62×10−66	34.5
LDL-C	19	47603609	*LIPE*	rs34052647	G	0.96	0.43 (0.098)	9.65×10−6	0	0.999	NA	NA	NA
HDL-C	1	202392868	*REN*	rs11571087	T	0.01	0.28 (0.060)	2.58×10−6	53	0.03	0.006 (0.006)	0.89	13.1
HDL-C	3	149905771	*AGTR1*	rs12721308	G	0.01	0.27 (0.060)	4.95×10−6	0	0.001	NA	NA	NA
HDL-C	15	56512084	*LIPC*	rs8034802	A	0.48	0.07 (0.012)	1.82×10−8	2.5	0.28	0.03 (0.002)	4.86×10−43	18.2
HDL-C	16	55550825	*CETP*	rs3764261	A	0.31	0.08 (0.012)	3.42×10−11	63.2	0.31	0.07 (0.002)	8.45×10−270	65.3
TG	11	116169250	*APOA5*	rs10750097	G	0.4	0.14 (0.020)	2.14×10−12	0	0.21	0.07 (0.004)	4.16×10−93	52.9

Chr chromosome, BP base pair, RAF risk allele frequency, SE standard error.

#### African Americans

We found five independent loci that were associated with TC at the genome-wide significance threshold. Four of these signals were SNPs lying within previously described loci: *LDLR* (rs6511720, *p* = 1.4×10^−13^); *CELSR2* (rs12740374, *p* = 4.4×10^−13^); *APOE* (rs389261, *p* = 2.1×10^−11^) and *PCSK9* (rs11806638, *p* = 2.00×10^−9^), while one signal was a novel SNP within *ICAM1* (rs5030359, *p* = 5.2×10^−9^). Three SNPs in the previously known loci, *CELSR2* (rs12743074, *p* = 1.9×10^−17^), *APOE* (rs389261, *p* = 1.0×10^−12^) and *PCSK9* (rs11800231, *p* = 1.0×10^−10^) reached genome-wide significance for association with LDL-C. We also identified a novel signal within *ICAM1* (rs5030359, *p* = 1.1×10^−7^) that is associated with LDL-C in African Americans at array-wide significance. Genome-wide significant association with HDL-C was observed for three SNPs in previously identified loci within *CETP* (rs17231520 *p* = 2.0×10^−46^), LPL (rs13702 *p* = 1.3×10^−9^) and *LIPC* (rs2070895 *p* = 4.2×10^−8^). Of the array-wide significant loci, rs3211938 within *CD36* (*p* = 3.1×10^−7^) has been previously described to be associated with HDL-C in a candidate gene study of 2,020 African Americans [25] but had not previously been identified in a large-scale genomic study. For TG, we identified one association signal, rs12721054, within the previously reported *APOE* locus with TG with at genome-wide significance (*p* = 1.0×10^−21^).

#### Hispanics

Genome-wide significant association with HDL-C was observed for two SNPs in previously identified loci within *CETP* (rs3764261, *p* = 3.4×10^−11^) and *LIPC* (rs8034802, *p* = 1.8×10^−8^). For TG, we identified one genome-wide signal within the previously reported *APOA5* locus (rs10750097, *p* = 2.1×10^−12^). Genome-wide significant association for TC and LDL-C was not observed in our Hispanic populations.

#### East Asians

In East Asians, the rs662799 variant within *ZNF259/APOA5* was significantly associated with TG (*p* = 1.6×10^−13^). The opposite allele of the same SNP was study-wide significantly associated with HDL-C. Genome-wide or study-wide significant genetic association was not observed for LDL-C or TC in our East Asian populations.

### Independent signals within single genetic loci in African Americans

The current investigation using the IBC array included rare SNPs at candidate loci collected in sequencing data from Europeans and Africans and dense genotyping, which can potentially be used to identify independent signals for lipids within genes at known or novel loci. We repeated association studies conditioning on the lead SNP in 23 loci with *P*<1.0×10^−5^. After Bonferroni correction for the number of SNPs at each candidate gene locus, we found independent lipids signals at the *LDLR*, *APOE*, *PCSK9* and *APOB* loci for TC, at the *APOE*, *PCSK9*, *LDLR*, and *APOB* loci for LDL-C, at the *APOC1/APOE*, and *LPL* loci for TG and at the *CETP*, *LPL*, *CD36* and the *TRADD/LCAT* for HDL-C (Table 2).

**Table 2:** Loci with significant evidence of independent lipid association signals.

							Original signal		Second signal		Third signal		
Trait	Gene	SNP	Chr	BP	Risk Allele	RAF	Beta (SE)	*P*	Beta (SE)	*P*	Beta (SE)	*P*	r2 with lead SNP
TC	*LDLR*	rs6511720	19	11063306	G	0.86	0.18 (0.024)	1.39×10−13					
		rs17242787	19	11063460	T	0.98	0.35 (0.059)	4.67×10−9	0.37 (0.059)	2.44×10−10			0.004
	*APOE*	rs389261	19	50112183	A	0.25	0.13 (0.0120)	2.07×10−11					
		rs283813	19	50081014	T	0.67	0.09 (0.018)	3.60×10−7	0.09 (0.018)	1.30×10−6			0.001
		rs12721054	19	50114427	A	0.88	0.15 (0.027)	4.75×10−8			0.10 (0.027)	1.85×10−4	0.025
	*PCSK9*	rs11806638	1	55290748	C	0.68	0.11 (0.018)	1.99×10−9					
		rs505151	1	55301775	G	0.24	0.11 (0.019)	7.50×10−9	0.09 (0.0120)	9.78×10−6			0.085
	*APOB*	rs12720826	2	21119200	T	0.87	0.13 (0.025)	7.84×10−8					
		rs562338	2	21141826	G	0.4	0.09 (0.017)	2.00×10−7	0.07 (0.018)	1.20×10−4			0.054
LDL-C	*APOE*	rs389261	19	50112183	A	0.25	0.14 (0.020)	1.03×10−12					
		rs283813	19	50081014	T	0.67	0.12 (0.018)	7.27×10−12	0.12 (0.018)	3.30×10−11			0.001
		rs166907	19	50078695	G	0.12	0.08 (0.026)	2.92×10−3			0.16 (0.031)	1.96×10−7	0.001
	*PCSK9*	rs11800231	1	55290528	G	0.82	0.14 (0.022)	1.02×10−10					
		rs505151	1	55301775	G	0.24	0.12 (0.019)	1.91×10−10	0.10 (0.020)	2.15×10−7			0.12
		rs1165287	1	55292800	A	0.25	0.09 (0.02)	7.42×10−6			0.08 (0.021)	1.17×10−4	0.08
	*LDLR*	rs17248720	19	11059187	C	0.73	0.11 (0.019)	7.13×10−10					
		rs6511720	19	11063306	T	0.86	0.19 (0.024)	7.91×10−15	0.15 (0.030)	2.93×10−7			0.251
		rs17242787	19	11063460	T	0.98	0.31 (0.06)	1.76×10−7			0.33 (0.062)	8.12×10−8	0.071
	*APOB*	rs562338	2	21119200	G	0.4	0.09 (0.017)	6.54×10−8					
		rs12720826	2	21119200	T	0.87	0.13 (0.025)	7.84×10−8	0.11 (0.026)	1.30×10−4			0.054
HDL-C	*CETP*	rs17231520	16	55553328	A	0.07	0.19 (0.013)	2.03×10−46					
		rs4783961	16	55552395	A	0.44	0.09 (0.007)	6.08×10−40	0.06 (0.007)	2.83×10−20			0.165
		rs7499892	16	55564091	C	0.62	0.07 (0.007)	7.27×10−24			0.04 (0.006)	6.40×10−9	0.078
	*LPL*	rs13702	8	19868772	C	0.51	0.04 (0.007)	1.34×10−9					
		rs3289	8	19867472	T	0.93	0.07 (0.013)	5.07×10−8	0.06 (0.013)	2.70×10−5			0.047
	*CD36*	rs3211938	7	80138385	G	0.09	0.06 (0.012)	3.09×10−7					
		rs3211849	7	80121259	G	0.54	0.02 (0.007)	5.50×10−3	0.03 (0.007)	1.33×10−5			0.118
	*TRADD/LCAT*	rs35673026	16	66534352	T	0.004	0.28 (0.059)	2.40×10−6					
		rs2233455	16	65765434	T	0.29	0.03 (0.007)	3.72×10−6	0.03 (0.007)	2.78×10−6			0
TG	*APOC1/APOE*	rs12721054	19	50114427	A	0.89	0.12 (0.013)	1.01×10−21					
		rs7258987	19	50124360	T	0.03	0.11 (0.024)	2.14×10−6	0.11 (0.024)	3.42×10−6			0.003
	*LPL*	rs328	8	19864004	C	0.93	0.08 (0.016)	1.74×10−7					
		rs3289	8	19867472	G	0.07	0.08 (0.016)	2.82×10−6	0.07 (0.016)	1.52×10−5			0.003

Chr chromosome, BP base pair, RAF risk allele frequency, SE standard error.

Three loci harbored two independent signals at genome-wide significance. The alleles rs6511720-G (risk allele frequency [RAF] = 0.86) and rs17242787-T (RAF = 0.98) within the *LDLR* gene showed association with TC with a p-value of 1.04×10^−13^ and 4.7×10^−9^ respectively in the original analyses. After conditioning on rs6511720-G, the *p* value for rs17242787-T remained significant (*p* = 2.4×10^−10^). Also for LDL-C, we found two independent genome-wide significant signals within the *APOE* locus: rs389261-A (RAF = 0.25) and rs283813-T (RAF = 0.67). Furthermore, the SNPs rs17231520-A (RAF = 0.07) and rs4783961-A (RAF = 0.44) within the *CETP* gene were both strongly associated with HDL-C and after conditioning on the lead signal, the secondary signal remained significant with *p* = 2.8×10^−20^. Interestingly, the newly identified *CD36* locus also harbored two independent signals, with the second signal showing association with locus-wide significance. The *r^2^* between the two SNPs in HapMap-YRI was 0.118.

### Replication

In order to confirm putative novel signals, we carried out *in silico* follow-up of ten SNPs within novel loci and previously unreported SNPs within known lipid-associated loci (*P*<1.0×10^−5^) in six African American studies, comprising together 7,000 samples. Only HeartSCORE was genotyped using the IBC array and provided association results for all SNPs. All other replication studies contributed association results for up to seven genotyped and imputed SNPs. Imputed SNPs were only included in the study when passing the 95% confidence threshold. Combined meta-analysis of the discovery and replication studies led to genome-wide significant signals at the *CD36* locus (*p* = 13.5×10^−12^; Table 3) for association with HDL-C. A signal within *ACADL* was not significant after meta-analysis of the discovery and replication studies. However, the direction of effect was consistent with our discovery dataset in three of six studies, so it is possible that the signal has a weak effect and the locus is undetectable due to limited statistical power. Also, previously unidentified signals in known lipid loci showed genome-wide significant association in the combined discovery and replication meta-analysis: rs11806638 within *PCSK9* was found to be associated with TC; rs389261 within *APOE* was associated with LDL-C levels; rs17231520 within the *CETP* locus and rs35673026 within the *LCAT* locus were found to be associated with HDL-C; and rs12721054 within *APOE* was associated with TG levels (Table 3).

**Table 3:** Replication results of nine signals in 7,000 African Americans.

Novel loci					Discovery Set (N = 7,657)		Replication Set (N = 7,000)		Combined Set (N = 14,657)	
Trait	Candidate Gene	SNP	Risk allele	RAF	Beta (SE)	P	Beta (SE)	P	Beta (SE)	P
TC	*ACADL*	rs6739874	T	0.05	0.17 (0.038)	4.65×10−6	0.01 (0.043)	0.86	0.11 (0.029)	1.72×10−4
TC	*ICAM1*	rs5030359	G	0.99	0.57 (0.098)	5.22×10−9	0.48 (0.295)	0.10	0.44 (0.095)	4.33×10−6
LDL-C	*ICAM1*	rs5030359	G	0.99	0.51 (0.096)	1.09×10−7	0.47 (0.264)	0.08	0.40 (0.089)	8.74×10−6
HDL-C	*CD36*	rs3211938	G	0.09	0.06 (0.012)	3.09×10−7	0.06 (0.013)	3.12×10−6	0.06 (0.009)	3.49×10−12

unidentified signals in previously known loci					Discovery Set (N = 7,657)		Replication Set (N = 7,000)		Combined Set (N = 14,657)	
Trait	Candidate Gene	SNP	Risk allele	RAF	Beta (SE)	P	Beta (SE)	P	Beta (SE)	P
TC	*PCSK9*	rs11806638	C	0.68	0.11 (0.018)	1.99×10−9	0.15 (0.029)	3.78×10−7	0.12 (0.016)	2.38×10−14
LDL-C	*APOE*	rs389261	A	0.25	0.14 (0.020)	1.03×10−12	0.13 (0.028)	4.43×10−6	0.14 (0.016)	1.70×10−17
HDL-C	*CETP*	rs17231520	A	0.07	0.19 (0.013)	2.03×10−46	0.18 (0.021)	2.35×10−17	0.18 (0.011)	1.70×10−62
HDL-C	*LCAT*	rs35673026	T	0.005	0.28 (0.059)	2.40×10−6	0.05 (0.12)	0.71	0.24 (0.053)	9.06×10−6
TG	*APOE*	rs12721054	A	0.89	0.12 (0.013)	1.01×10−21	0.05 (0.008)	4.71×10−13	0.07 (0.007)	1.31×10−28

RAF risk allele frequency, SE standard error.

### Comparison of lipid loci in African Americans to Europeans

Utilizing the results of each of the meta-analyses from the three available ethnicities, we sought to refine localization of known lipid signals or reveal novel independent signals within known loci based upon differential LD (see Table 1). The dense genotyping within each locus on the IBC array enabled detailed comparisons of loci that harbored array-wide significant SNPs in Africans Americans, Hispanics and East Asians as well as in the IBC meta-analysis of up to 61,636 individuals of Europeans ancestry [21] (see Table 1 and Table S3).

The strongest signal for HDL-C in African Americans is rs17231520 within *CETP* (*p* = 2.0×10^−46^; Table 1). This SNP is associated with HDL-C in the same direction in Europeans with *p* = 3.3×10^−4^. However, in Europeans there is less power to detect this signal at array-wide significance, as the MAF in Europeans is only 0.2% (versus 7% in African Americans) and was screened out in many European studies for the IBC meta-analysis. Furthermore, rarer variants are often not correctly clustered optimally during QC, making them less likely to pass the standard quality control (including genotyping threshold or HWE check). This is also observed for the most strongly associated SNPs within *CD36* (rs3211938) and *LCAT* (rs35673026) for HDL-C in African-Americans, as they show the same direction of effect in Europeans, but do not reach significance, given low MAF and absence in the majority of European studies for IBC meta-analysis. For two loci, *LIPC* and *LPL*, the strongest associated SNP in African Americans for HDL-C was the same or among the most highly associated SNPs in Europeans. Also, for the LDL-C-associated loci *CELSR2*, *APOB*, *APOH* and *LDLR*, the strongest signals in African Americans did overlap or represented similar signals that were highly associated with LDL-C in Europeans. The newly identified SNP for LDL-C, rs5030359 within *ICAM1*, has an observed MAF of 0.8% in African Americans and 0.2% in Europeans. In Europeans, this SNP is not associated with LDL-C (*p* = 0.3231), but the SNP is only present in very few European studies that are included in the IBC meta-analysis. The most associated signals within *PCSK9* and *APOE* in African Americans are different, independent signals compared to the most associated SNPs within these loci in Europeans. Again, both signals are common in African Americans and have very low frequencies in Europeans: MAF for SNPs in *PCSK9* and *APOE* are 17% and 25% in African Americans and 0.5% and 0.1% in Europeans respectively.

Among the array-wide statistically significant loci that were associated with TG in African Americans, three SNPs within *GCKR, LPL* and *APOA5* were the same as or amongst the most highly associated SNPs in Europeans. SNP rs12721054 in *APOE* appeared to be a novel independent signal for TG in African Americans. This SNP showed an opposite effect in European-derived cohorts, although it was observed rarely in the meta-analysis of European populations (MAF = 0.2%) [13].

For TC, we observed the same pattern as for other lipid traits. The strongest associated SNPs within loci associated with TC overlapped with the same signals in Europeans (SNPs within *CELSR2*, *APOB*, *LDLR* and *APOE*), or were independent signals in African Americans that could not be replicated in Europeans because of low frequency (*PCSK9*, *ACADL* and *ICAM1*).

### Direction of effect concordance with lead SNPs identified in European populations

Direction of effect across different ethnicities was studied for 28 previously established TC risk loci, 20 LDL-C loci, 24 HDL-C loci, and 21 TG associated loci. Not all SNPs passed the initial quality control, so number of investigated SNPs differed by trait and ethnicity (Table S3).

Concordance in direction of effect was observed for 21/27 (*p* = 0.033), 15/20 (*p* = 0.102), 16/23 (*p* = 0.176) and 19/21 (*p* = 0.004) association signals for TC, LDL-C, HDL-C and TG, respectively, between Europeans and African Americans; 23/28 (*p* = 0.011), 16/20 (*p* = 0.047), 21/23 (*p* = 0.002) and 19/21 (*p* = 0.004) SNPs were concordant in direction of effect for TC, LDL-C, HDL-C and TG respectively between Europeans and Hispanics. Finally, 17/24 SNPs for TC (*p* = 0.140), 11/16 SNPs for LDL-C (*p* = 0.279), 16/29 SNPs for HDL-C (*p* = 0.196) and 17/21 (*p* = 0.035) SNPs for TG were concordant between Europeans and East Asians (Table S3).

### Genetic risk score analysis

To study whether we could find elevated lipid levels in multi-ethnic samples with cumulative numbers of risk alleles that were previously found to be associated in Europeans, we evaluated the contribution of the weighted genetic risk score for lipids in linear regression models adjusting for 10 PCs and compared the relative beta's ratios across quartiles of risk. We demonstrated a significant per quartile risk effect in African-Americans (ranging from *p*<10^−10^ for TG to *p*<10^−33^ for HDL-C), Hispanics (ranging from *p*<10^−l^ for LDL-C to *p*<10^−23^ for TC) and East Asians (ranging from *p*<0.02 for HDL-C to *p*<10^−6^ for TG) (see Table 4). Quartiles based on weighted risk alleles and lipid level distribution for each ethnicity is shown in Figure S1.

**Table 4:** Risk score analysis of lipid profile in multiethnic populations, using weighted score of known lipid SNPs.

African Americans				
Trait	TC	LDL-C	HDL-C	TG
Beta (SE)	0.12 (0.011)	0.08 (0.01)	0.05 (0.004)	0.02 (0.004)
P	7.57×10−23	5.45×10−15	3.13×10−33	4.83×10−10
Quartiles of Risk Alleles				
Q1 Beta (SE)	ref	ref	ref	ref
Q2 BETA (SE)	0.12 (0.034)	0.12 (0.033)	0.05 (0.014)	0.06 (0.017)
P	2.99×10−04	3.07×10−04	1.23×10−04	8.04×10−04
Q3 BETA (SE)	0.25 (0.035)	0.16 (0.034)	0.10 (0.014)	0.05 (0.017)
P	1.88×10−12	1.88×10−06	2.45×10−12	2.62×10−03
Q4 BETA (SE)	0.33 (0.036)	0.26 (0.034)	0.16 (0.013)	0.12 (0.017)
P	1.98×10−20	7.87×10−15	6.73×10−32	3.37×10−13

Hispanics				
Trait	TC	LDL-C	HDL-C	TG
Beta (SE)	0.17 (0.017)	0.08 (0.015)	0.07 (0.009)	0.07 (0.009)
P	2.16×10−23	2.07×10−07	4.43×10−14	4.43×10−14
Quartiles of Risk Alleles				
Q1 Beta (SE)	ref	ref	ref	ref
Q2 BETA (SE)	0.09 (0.063)	0.06 (0.046)	0.06 (0.019)	0.05 (0.036)
P	0.15	0.19	0.005	0.15
Q3 BETA (SE)	0.26 (0.063)	0.09 (0.048)	0.08 (0.02)	0.02 (0.033)
P	2.78×10−05	0.06	9.65×10−05	0.56
Q4 BETA (SE)	0.47 (0.056)	0.24 (0.047)	0.166	0.18 (0.034)
P	6.42×10−17	2.65×10−07	3.42×10−16	2.68×10−07

East Asians				
Trait	TC	LDL-C	HDL-C	TG
Beta (SE)	0.0642 (0.0288)	0.0137 (0.0272)	0.105 (0.044)	0.088 (0.0186)
P	0.03	0.62	0.02	2.29×10−06
Quartiles of Risk Alleles				
Q1 Beta (SE)	ref	ref	ref	ref
Q2 BETA (SE)	0.32 (0.097)	−0.03 (0.084)	0.09 (0.039)	0.08 (0.059)
P	8.37×10−04	0.74	0.02	0.17
Q3 BETA (SE)	0.22 (0.094)	−0.08 (0.092)	0.13 (0.038)	0.10 (0.063)
P	0.02	0.37	6.82×10−04	0.1
Q4 BETA (SE)	0.25 (0.092)	0.05 (0.085)	0.19 (0.039)	0.27 (0.058)
P	0.007	0.53	2.27×10−06	2.39×10−06

ref reference group, SE standard error.

## Discussion

The current study reports a meta-analysis of lipid association studies in African Americans, Hispanics and East Asians using the IBC array, and has identified two novel loci associated with TC and LDL-C levels (rs5030359 in *ICAM1*) and HDL-C levels (rs3211938 in *CD36*) in African Americans. Additionally, we have uncovered multiple independent association signals within established lipid loci, demonstrating the value of dense SNP genotyping to uncover genetic variation associated with lipid levels. Furthermore, we have evaluated the impact of established SNPs, previously associated with lipids in Europeans populations, on lipid levels in three additional populations, showing that many known association signals for lipids span across ethnicities.

### CD36

This study shows association between the nonsense coding variant rs3211938-G in *CD36* and HDL-C levels at conventional genome-wide significance for African Americans (*p*<5×10^−9^). This SNP has previously been reported to be associated with increased HDL-C levels (*p* = 0.00018), decreased TG levels (*p* = 0.0059) and protection against metabolic syndrome (*p* = 0.0012) in a candidate gene study including 2,020 African Americans that did not overlap with samples in our meta-analyses [25]. Also, a variant within *CD36* was associated with LDL levels in two small studies [26], [27]. The CD36 finding is present in an accompanying paper [52] from the wider NHLBI CARe lipid studies which essentially uses the same discovery cohorts for African Americans that we present here although our analysis differs in that (a) it screened out related individuals (b) it takes additional covariates into account through the use of the three multivariate models and (c) our analysis filtered more stringently on *I* ^2^ and (d) we replicated these findings in additional studies.

CD36, which is present on gustatory, olfactory and intestinal epithelial cells, is involved in the orosensory perception of fatty acids [28], [29]. Also, lipid ingestion affects lingual CD36 expression in mice [30]. Therefore, CD36 may influence fat intake, and hence, serum lipid levels. SNPs within *CD36*, other than the one we found in this study, were linked to obesity in a case-control study [31]. However, this finding could not be replicated in a larger cohort [32]. In mouse models, CD36 deficiency impairs intestinal lipid secretion and results in hypertriglyceridemia [33] and others show that CD36 deficiency rescues lipotoxic cardiomyopathy [34].

CD36 is an integral membrane protein found on the surface of many cell types and binds many ligands including oxidized lipid proteins [35], [36], long-chain fatty acids [37] and erythrocytes that are parasitized with the malaria parasite *Plasmodium falciparum* [38]. The rs3211938-G variant is nearly absent in Europeans and Asians and shows a signature of selection in African Americans and some African populations [39], [40]. Additionally, rs3211938-G has been shown in previous studies to be associated with CD36 deficiency and with susceptibility to malaria, although this has not been confirmed in other studies [41], [42].

### *ICAM1*

The rs5030359 variant in *ICAM1*, is observed in this study to be associated with TC and LDL-C at conventional genome-wide significance. *ICAM1* encodes a cell surface glycoprotein that is typically expressed on endothelial cells and cells of the immune system [43]. However, rs5030359 maps to a gene-dense region (Figure 2b), so it cannot be excluded that there is another gene underlying the signal. The rs5030359 variant is ∼800 kb downstream of a previously identified lipids signal within the *LDLR* region, but conditional analyses showed that the two loci are independent. Using fine-mapping in non-African populations to point to the most likely gene underlying the signal, is not possible as the SNP is very rare in Europeans, with a MAF of 0.002, and absent in our Hispanic and East Asian populations. Previously, common variants within *ICAM1* were found to be associated with soluble ICAM1 (sICAM1) concentrations in Europeans [44], [45]. sICAM1 has been associated with several common diseases such as diabetes, heart disease, stroke, and malaria [46], [47]. sICAM1 levels were associated with progression of carotid intima media thickness in young adults [48], [49] and in asymptomatic dyslipidaemia subjects [50]. Additionally, sICAM1 levels were found to be higher in Europeans than in Africans [49].

### Differences in signals within lipid loci in multiple ethnicities

We were able to use the dense SNP genotyping in loci on the IBC array to analyze and compare lipid-associated loci, particularly between African Americans and Europeans. Our analyses showed multiple examples of signals that were associated with lipid levels in one ethnicity but not another (Table 1).

First, some of the strongest associated SNPs in one ethnicity may be rare or absent in other ethnicities. This is a well-established phenomenon, e.g., truncation mutations in *PCSK9* that are of low frequency in African Americans and absent in individuals of European origin, that result in a robust reduction in LDL-C levels and coronary heart disease risk [51], [52]. In this study we find that the majority of the observed discrepancies across ethnicities in association of SNPs with lipid traits can be attributed to differences in allele frequency. For example, rs3211938 in *CD36* is much more highly associated with HDL-C in African Americans (*p* = 1.8×10^−11^) than in Europeans (*p* = 0.08) with a large discrepancy in RAFs (7% vs. 0.2%).

In other loci, the strongest associated polymorphisms varied across populations, for example in the *BUD13/ZNF259/APOA5* region (Table S3, Figure S2). In theory these regions could be excellent candidates for fine-mapping, but our efforts and association results could not narrow down the loci. When conducting meta-analyses across multiple ethnicities we observed that the stronger p-value association typically tracked with the higher heterogeneity *I^2^* values (Figure S3). This high *I^2^* suggests high heterogeneity, but it could also be the effect of low sample sizes of the combined cohorts (especially for Hispanics and East Asians).

One limitation of this study is the sample size available particularly the Hispanic and the East Asian available samples and this obviously limited our ability to find new signals in these populations and to replicate many previously established lipid signals. Also, not all previously described signals for lipids were present on the IBC array, as the array was designed to densely cover genes regions, rather than the whole genome. However, using this approach we did find signals for lipids that remained uncovered using the genome-wide association approach, as both rs5030359 within *ICAM1* and rs3211938 within *CD36* were not present on conventional genome-wide arrays.

In conclusion, we performed dense genotyping of ∼2,000 candidate genes in 7,657 African Americans, 1,315 Hispanics and 841 East Asians using IBC 50K SNP genotyping array and we found and confirmed two novel signals for lipids by replication in 7,000 African Americans. Additionally we evaluated the effect of SNPs established in European populations on lipid levels in multi-ethnic populations and show that most known lipid association signals span across ethnicities. However, differences between populations, especially differences in allele frequency, can be leveraged to identify novel signals.

## Supporting Information

Figure S1 Quartiles based on the number of weighted risk alleles and lipid level distribution.
(TIF)

Figure S2 Association results of the *BUD13/ZNF259/APOA5* regions with TG in multiple ethnicities. Beta's of SNPs are shown in the *BUD13/ZN259/APOA5* region from association results in each ethnicity separately. −logP-value and I^2^ are from multi-ethnic meta-analyses. Squares mark the strongest association signals per ethnicity. The three independent signals in Europeans are depicted in green, the top signal in African Americans is shown in blue and Hispanics and East Asian meta-analyses results are in red and yellow respectively.
(PDF)

Figure S3 Correlation between −logP-value and I^2^ in the *BUD13/ZN259/APOA5* region for TG association results.
(TIF)

Supporting Information S1 Supplementary acknowledgments.
(DOCX)

Table S1 Characteristics of studies contributing to the multi-ethnic IBC lipids meta-analyses.
(XLSX)

Table S2 Summary statistics for covariates in participating studies providing individual-level data.
(XLSX)

Table S3 Association results for known lipids loci in Europeans, African-Americans, Hispanics and East Asians.
(XLSX)

Table S4 Loci associated with lipid traits in individuals of African American, Hispanic and East Asian origin (model 2, model 3).
(XLSX)
