# Supplementary figures and images for: Gene-Centric Meta-Analysis of Lipid Traits in African, East Asian and Hispanic Populations
Source: PLoS One. 2012 Dec 7;7(12):e50198. doi: 10.1371/journal.pone.0050198 (PMC3517599; doi:10.1371/journal.pone.0050198)

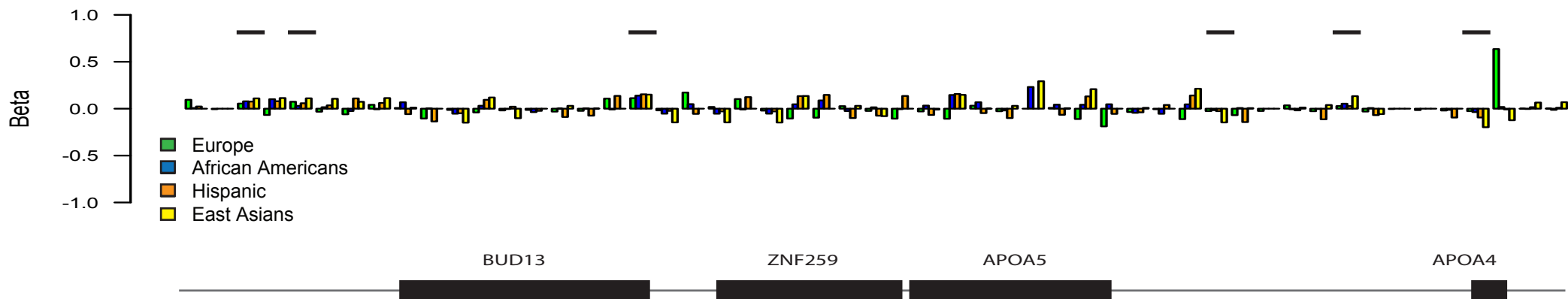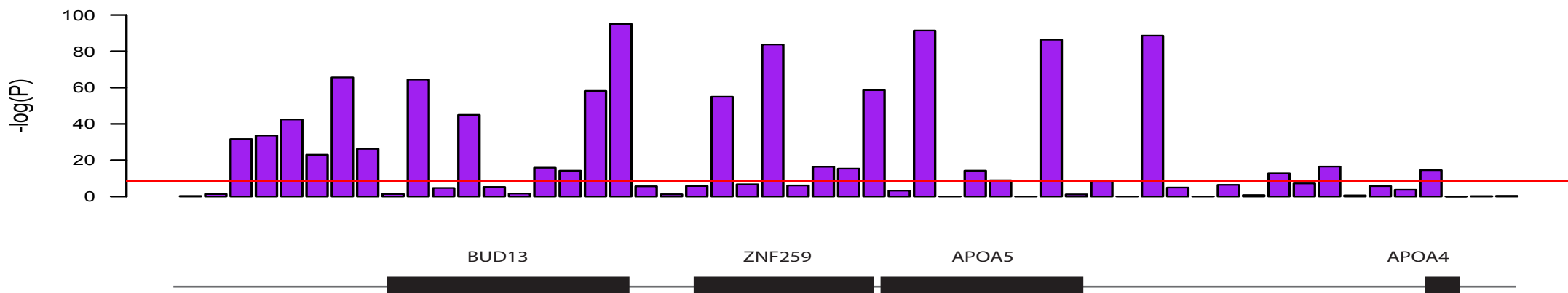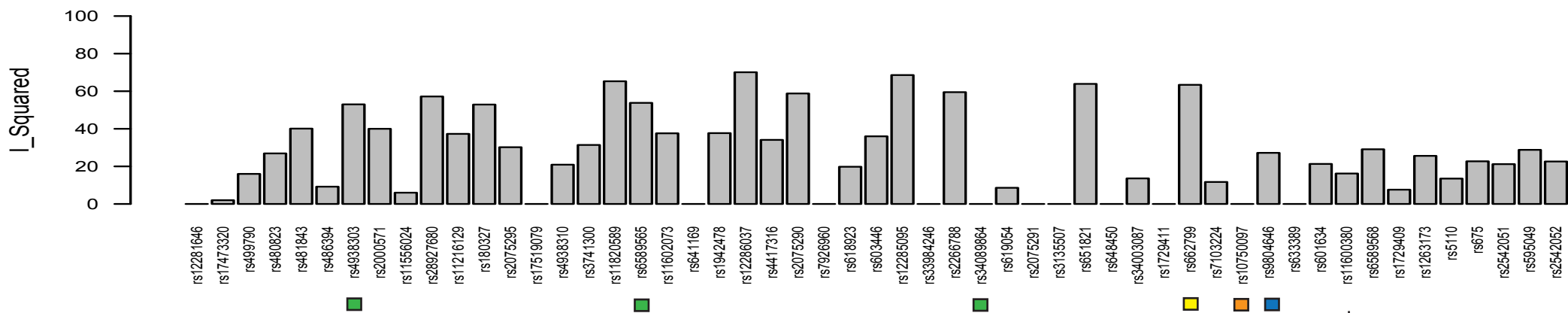

Supplement: Figure S2 — Association results of the BUD13/ZNF259/APOA5 regions with TG in multiple ethnicities. Beta's of SNPs are shown in the BUD13/ZN259/APOA5 region from association results in each ethnicity separately. −logP-value and I2 are from multi-ethnic meta-analyses. Squares mark the strongest association signals per ethnicity. The three independent signals in Europeans are depicted in green, the top signal in African Americans is shown in blue and Hispanics and East Asian meta-analyses results are in red and yellow respectively. (PDF) [file pone.0050198.s002.pdf]

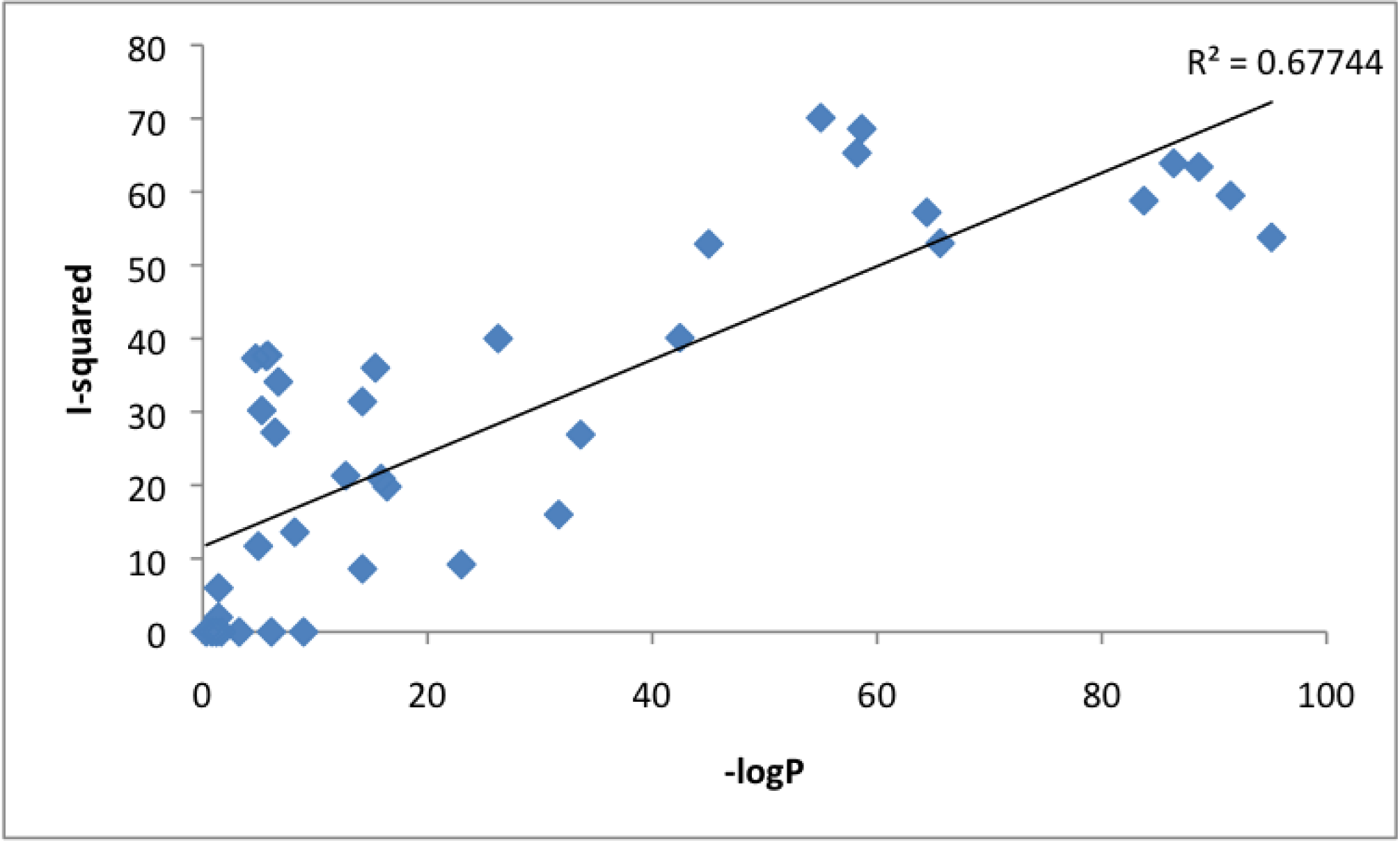

Supplement: Figure S3 — Correlation between −logP-value and I2 in the BUD13/ZN259/APOA5 region for TG association results. (TIF) [file pone.0050198.s003.tif]
