# Supplementary material for: Gene-Centric Meta-Analysis of Lipid Traits in African, East Asian and Hispanic Populations
Source: PLoS One. 2012 Dec 7;7(12):e50198. doi: 10.1371/journal.pone.0050198 (PMC3517599; doi:10.1371/journal.pone.0050198)
Supplement: Supporting Information S1 — Supplementary acknowledgments. (DOCX) [file pone.0050198.s004.docx]

Supplemental Acknowledgements

**ARIC:** The Atherosclerosis Risk in Communities Study is carried out as a collaborative study supported by National Heart, Lung, and Blood Institute contracts N01-HC-55015, N01-HC-55016, N01-HC-55018, N01-HC-55019, N01-HC-55020, N01-HC-55021, N01-HC-55022, R01HL087641, R01HL59367, R01HL086694 and RC2 HL102419; National Human Genome Research Institute contract U01HG004402; and National Institutes of Health contract HHSN268200625226C.  The authors thank the staff and participants of the ARIC study for their important contributions.  Infrastructure was partly supported by Grant Number UL1RR025005, a component of the National Institutes of Health and NIH Roadmap for Medical Research.

**CARDIA:** The Coronary Artery Risk Development in Young Adults (CARDIA) study is funded by contracts N01-HC-95095, N01-HC-48047, N01-HC-48048, N01-HC-48049, N01-HC-48050, N01-HC-45134, N01-HC-05187, N01-HC-45205, and N01-HC-45204 from the National Heart, Lung, and Blood Institute to the CARDIA investigators.

**CHS:** The Cardiovascular Health Study research was supported by NHLBI contracts N01-HC-85239, N01-HC-85079 through N01-HC-85086; N01-HC-35129, N01 HC-15103, N01 HC-55222, N01-HC-75150, N01-HC-45133, NHLBI contract HHSN268200960009C, and NHLBI grants HL080295, HL075366, HL087652, HL105756 with additional contribution from NINDS. Additional support was provided through AG-023629, AG-15928, AG-20098, and AG-027058 from the NIA. See also http://www.chs-nhlbi.org/pi.htm.

**JHS:** This research from the Jackson Heart Study is supported by NIH contracts N01-HC-95170, N01-HC-95171, and N01-HC-95172 provided by the National Heart, Lung, and Blood Institute and the National Center for Minority Health and Health Disparities.

**MESA:** Multi-Ethnic Study of Atherosclerosis (MESA) was supported by the following: University of Washington (N01-HC-95159), University of California, Los Angeles (N01-HC-95160), Columbia University (N01-HC-95161), Johns Hopkins University (N01-HC-95162, N01-HC-95168), University of Minnesota (N01-HC-95163), Northwestern University (N01-HC-95164), Wake Forest University (N01-HC-95165), University of Vermont (N01-HC-95166), New England Medical Center (N01-HC-95167), Harbor-UCLA Research and Education Institute (N01-HC-95169), Cedars-Sinai Medical Center (R01-HL-071205) and University of Virginia.

**SHARE:** SHARE (Study of Health and Atherosclerosis risk in multiple Ethnicities) was funded by Canadian Institutes of Health Research (CIHR) (MOP-13430, MOP-79523, CTP-79853), the Heart and Stroke Foundation of Ontario (NA-6059, T-6018, PRG-4854), Genome Canada through Ontario Genomics Institute and the Pfizer Jean Davignon Distinguished Cardiovascular and Metabolic Research Award.

**WHI**: The WHI program is funded by the National Heart, Lung, and Blood Institute, National Institutes of Health, U.S. Department of Health and Human Services through contracts N01WH22110, 24152, 32100-2, 32105-6, 32108-9, 32111-13, 32115, 32118-32119, 32122, 42107-26, 42129-32, and 44221.

FamHS (Family Heart Study) – The FamHS is funded by a NHLBI grant 5R01HL08770003, and NIDDK grants 5R01DK06833603 and 5R01DK07568102. We also thank all of the participants and staff of the FamHS project.

GeneSTAR: GeneSTAR was supported by NIH grants through the National Institute of Nursing Research (NR0224103) and the National Heart, Lung, and Blood Institute (HL58625-01A1, HL59684, HL071025-01A1, U01HL72518, and HL087698); and by M01-RR000052 to the Johns Hopkins General Clinical Research Center.

HANDLS: This research was supported by the Intramural Research Program of the NIH, National Institute on Aging and the National Center on Minority Health and Health Disparities (project # Z01-AG000513 and human subjects protocol # 2009-149). Data analyses for the HANDLS study utilized the high-performance computational capabilities of the Biowulf Linux cluster at the National Institutes of Health, Bethesda, Md. (<http://biowulf.nih.gov>).

HealthABC: Health ABC was funded by the National Institutes of Aging. This research was supported by NIA contracts N01AG62101, N01AG62103, and N01AG62106. The genome-wide association study was funded by NIA grant 1R01AG032098-01A1 to Wake Forest University Health Sciences and genotyping services were provided by the Center for Inherited Disease Research (CIDR). CIDR is fully funded through a federal contract from the National Institutes of Health to The Johns Hopkins University, contract number HHSN268200782096C. This research was supported in part by the Intramural Research Program of the NIH, National Institute on Aging.

HeartSCORE: The HeartSCORE project is funded by the Pennsylvania Department of Health (Contract ME-02-384) and by the NIH (grant number R01: HL084613). We also thank Amy Beto, Mary Catherine Coast, Jowanda Green, and Lee Ann McDowell for their ongoing support for Heart SCORE.

SIGNET: SIGNET research was supported by R01 DK084350 (Sale), a cooperative agreement U01 NS041588 (George Howard, REGARDS) and the South Carolina Center of Biomedical Research Excellence (COBRE) for Oral Health P20-RR-17696 (project PI Fernandes) from the National Institutes of Health, and the W.M. Keck Foundation (Garvey, Project SuGAR).
